# 

**Published:** 1898-06

**Authors:** 


### CONTENTS.

COMMUNICATIONS.

Can Pyorrhea Alveolaris Be Cured? ... 251
Cataphoresis vs. The Direct Application of the Galvanic Current for Obtunding Sensitive Dentine and How to Exclude Both ... 255
The Need of Dental Instruction in Medical Schools ... 261
Dental Faculties in Medical Schools ... 264
Trigeminous Reflexes ... 267
The History of General Anæsthesia ... 269

PROCEEDINGS.

Changes in the Constitution of the Southern Dental Association, Making it a Branch of the National Dental Association ... 278
St. Augustine Meeting—Abstracts ... 279

SELECTIONS.

Restates the Law Relating to Malpractice ... 281
Dentition ... 282
Dentistry Practice—Kind of Certificates Required in Maryland ... 283
An Influence of the Law ... 284
Nerve Training ... 285
School Labor and Bodily Development ... 285
Quackery in Germany ... 286
On External Applications ... 287

COMMENCEMENTS.

Tennessee Medical College—Dental Department ... 287
University of Buffalo ... 288
Chicago College of Dental Surgery ... 288
Atlanta Dental College ... 290
Pennsylvania College of Dental Surgery ... 290
University of Maryland—Department of Dental Surgery. ... 291
Philadelphia Dental College ... 292
Baltimore College of Dental Surgery ... 294
Western Dental College ... 294
Indiana Dental College ... 295
Columbian Dental College ... 296
Western Reserve University, Dental College ... 296
Pittsburg Dental College ... 296
Meharry Medical College—Dental Department ... 297

NOTICES.

The Eleventh Annual Session of the Washington State Dental Society ... 298

EDITORIAL.

First Annual Meeting of the National Dental Association ... 299
The Annual Meeting of the National Association of Dental Faculties 300
A Beautiful Handle ... 300

